# Molecularly Imprinted Polymer Nanoparticles Enable Rapid, Reliable, and Robust Point-of-Care Thermal Detection of SARS-CoV-2

**DOI:** 10.1021/acssensors.2c00100

**Published:** 2022-04-13

**Authors:** Jake McClements, Laure Bar, Pankaj Singla, Francesco Canfarotta, Alan Thomson, Joanna Czulak, Rhiannon E. Johnson, Robert D. Crapnell, Craig E. Banks, Brendan Payne, Shayan Seyedin, Patricia Losada-Pérez, Marloes Peeters

**Affiliations:** † School of Engineering, Newcastle University, Merz Court, Claremont Road, Newcastle upon Tyne NE1 7RU, United Kingdom; ‡ Experimental Soft Matter and Thermal Physics (EST) Group, Department of Physics, Université Libré de Bruxelles, Boulevard du Triomphe CP223, Brussels 1050, Belgium; § MIP Diagnostics Ltd., The Exchange Building, Colworth Park, Sharnbrook, Bedford MK44 1LQ, United Kingdom; ∥ Faculty of Science and Engineering, Manchester Metropolitan University, John Dalton Building, Chester Street, Manchester M1 5GD, United Kingdom; ⊥ Department of Infection and Tropical Medicine, Royal Victoria Infirmary, Newcastle-upon-Tyne Hospitals NHS Foundation Trust, Newcastle upon Tyne NE1 4LP, United Kingdom; # Translational and Clinical Research Institute, Medical School, Newcastle University, Framlington Place, Newcastle upon Tyne NE1 7RU, United Kingdom

**Keywords:** biosensors, SARS-CoV-2, COVID-19, diagnostic testing, point-of-care testing, heat transfer method (HTM), molecularly imprinted polymer nanoparticles (nanoMIPs)

## Abstract

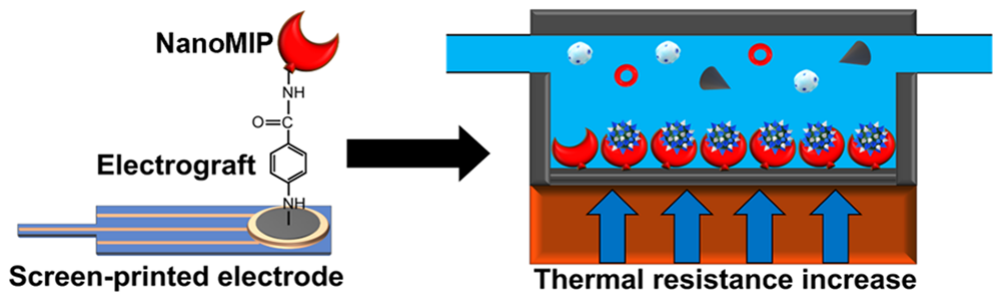

Rapid antigen tests are currently used for population screening of COVID-19. However, they lack sensitivity and utilize antibodies as receptors, which can only function in narrow temperature and pH ranges. Consequently, molecularly imprinted polymer nanoparticles (nanoMIPs) are synthetized with a fast (2 h) and scalable process using merely a tiny SARS-CoV-2 fragment (∼10 amino acids). The nanoMIPs rival the affinity of SARS-CoV-2 antibodies under standard testing conditions and surpass them at elevated temperatures or in acidic media. Therefore, nanoMIP sensors possess clear advantages over antibody-based assays as they can function in various challenging media. A thermal assay is developed with nanoMIPs electrografted onto screen-printed electrodes to accurately quantify SARS-CoV-2 antigens. Heat transfer-based measurements demonstrate superior detection limits compared to commercial rapid antigen tests and most antigen tests from the literature for both the alpha (∼9.9 fg mL^–1^) and delta (∼6.1 fg mL^–1^) variants of the spike protein. A prototype assay is developed, which can rapidly (∼15 min) validate clinical patient samples with excellent sensitivity and specificity. The straightforward epitope imprinting method and high robustness of nanoMIPs produce a SARS-CoV-2 sensor with significant commercial potential for population screening, in addition to the possibility of measurements in diagnostically challenging environments.

SARS-CoV-2 was first reported within Wuhan, China, in December 2019 and rapidly spread worldwide, leading to the World Health Organization (WHO) declaring it as a pandemic on 11th of March 2020. It is estimated that asymptomatic infection occurs in ∼20% of COVID-19 cases.^1−4^ Therefore, the identification of asymptomatic and presymptomatic cases is crucial to break the chain of SARS-CoV-2 transmission since asymptomatic individuals can possess equivalent viral loads to symptomatic cases.^5,6^ Reverse transcription-polymerase chain reaction (RT-PCR) tests are able to diagnose COVID-19 with high sensitivity (∼95%) and specificity (>95%).^7,8^ However, these tests are ineffective for general screening of the asymptomatic population due to the long turnaround time (∼1–2 days), high costs (∼$50 to the consumer),^9^ and shortages in facilities and trained personnel to conduct and analyze tests.^10,11^ Antigen tests offer portable and rapid (15–30 min) analysis that can be performed at the convenience of the individual.^12^ However, a critical review has shown that the majority of rapid antigen tests fail to meet the required sensitivity of the WHO (>80% for both symptomatic and asymptomatic cases) by a large margin, which has led to the withdrawal of several widely adopted tests from the market.^13,14^

For the detection of SARS-CoV-2, electroanalysis offers the prospect for scaling down analytical systems with features including low cost, rapid measurements, and high sensitivity.^15^ There are several reported electrochemical sensing platforms for SARS-CoV-2 in the literature, all of which utilize natural receptors such as antibodies or angiotensin-converting enzyme 2 (ACE2).^16−20^ The inherent drawback of these recognition elements is limited robustness such as the inability to withstand wide ranges of pH and temperature and a finite shelf-life. Furthermore, there are pressing concerns about batch-to-batch variation of antibodies due to lack of validation and the widespread use of animals in the antibody production process.^21^ These hurdles can be overcome by replacing natural recognition elements with artificial building blocks such as molecularly imprinted polymers (MIPs). MIPs are porous materials that contain high-affinity binding sites for their respective target molecule and have been successfully utilized as synthetic receptors for a variety of targets such as pathogens, biomarkers, and explosives.^22,23^ Different types of MIP-based sensors (*e.g.*, fluorescent, electrochemical, and resonant light) have also been utilized for the detection of numerous viruses.^24−26^ However, literature reports on MIP-based assays for SARS-CoV-2 detection are sparse with current sensor platforms generally using the whole SARS-CoV-2 spike protein or receptor binding domain (RBD) as a target, which leads to significant issues regarding scalability.^27−29^ MIP nanoparticles (nanoMIPs) are produced using an innovative solid-phase approach in which the solid phase acts as an affinity medium, enabling the collection of nanoMIPs with high affinity and homogeneous binding characteristics. NanoMIPs exhibit several clear advantages over conventionally produced MIPs such as excellent biocompatibility,^30^ fast binding kinetics,^31^ low manufacturing cost, and an automated production process.^32^ Previous studies have demonstrated that nanoMIPs consistently performed comparably or better than antibodies for the detection of many small molecules.^33^ Crucially, their short (2 h) and scalable production process is flexible and can be easily adapted to virtually any target of interest.^32^

Previously, we have incorporated nanoMIPs into a sensor platform, which utilizes the heat transfer method (HTM) for the detection of various targets such as protein biomarkers and antibiotics.^34−36^ The sensor platform measures the thermal resistance at the solid–liquid interface and possesses key advantages over many traditional diagnostic methods such as low-cost components, rapid and accurate detection, and simple quantitative analysis.^35,37^ Thermal detection technology can be implemented into portable devices to facilitate point-of-care testing. However, these point-of-care devices are yet to reach the mass market.^38^ Virus sensing using thermal detection remains unexplored due to the lack of high-affinity nanoMIPs, safety concerns regarding virus handling, and large sample volume requirement (several mL) for analysis. This work presents the first nanoMIP-based thermal assay for the fast (∼15 min) and accurate detection of SARS-CoV-2. The nanoMIPs are produced without biosafety risks by using only a small inactive virus fragment (∼10 amino acids) as the target. In addition to rivaling the affinity of antibodies, we demonstrate that nanoMIPs can withstand extremes of temperature (≤121 °C) and pH (5.5–8.5). This can lead to improved shelf-life and storage conditions compared to current antigen tests that can generally only be stored within narrow temperature ranges (15–30 °C).^39^ To improve the containment of COVID-19 outbreaks, there is also a drive toward developing sensors to rapidly detect SARS-CoV-2 in more diagnostically challenging environments such as wastewater. Contrary to antibodies, nanoMIPs are capable of functioning in more extreme environmental conditions and therefore offer the prospect for wastewater measurements without costly and laborious sample pretreatment.^40^

Using antigen-spiked solutions, we achieved superior detection limits (<10 fg mL^–1^) with nanoMIPs electrografted onto low-cost (<$0.1) screen-printed electrodes (SPEs) compared to commercial rapid antigen tests and many antigen tests from the literature.^41−47^ A miniaturized prototype of the thermal device (sample volume, 100 μL) was developed for rapid (∼15 min) clinical measurements, which exhibited excellent sensitivity and specificity. These favorable sensing capabilities paired with the high robustness of nanoMIPs create the potential for our sensor to be utilized for population screening, as well as for measurements in diagnostically challenging environments without the need for extensive sample pretreatment.^40^

## Results and Discussion

### NanoMIP and Functionalized Electrode Characterization

**Figure 1:**
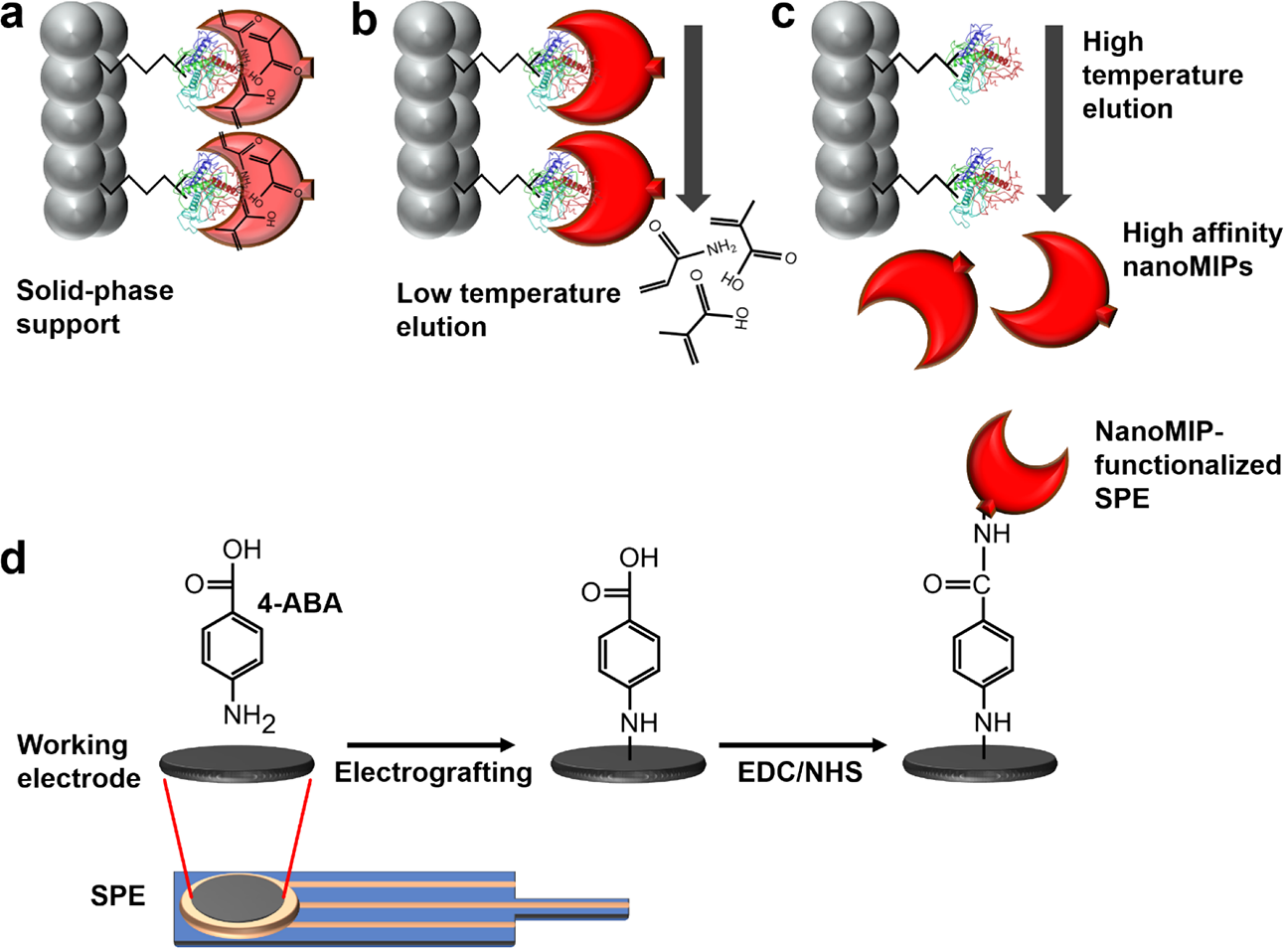
Schematic diagram of nanoMIP synthesis and SPE functionalization. (a) NanoMIP polymerization occurs around the epitope of the target, which is immobilized to a solid-phase support. (b) Low-temperature (20 °C) elution to remove low-affinity nanoMIPs and unreacted monomers. (c) Elevated temperature (60 °C) elution to collect high-affinity nanoMIPs. (d) NanoMIPs are covalently attached to an SPE surface by electrografting of 4-aminobenzoic acid (4-ABA), followed by a 1-ethyl-3-(3-dimethylaminopropyl) carbodiimide (EDC)/*N*-hydroxysuccinimide (NHS) coupling reaction.

The SARS-CoV-2 nanoMIPs were synthesized around the epitope of the target, which was attached to a solid-phase support (Figure 1a). This was followed by two elution steps to isolate and collect high-affinity nanoMIPs (Figure 1b,c). The target was an epitope (∼10 amino acids) of the RBD, which is the part of the spike protein that binds to the ACE2 receptor to gain entry into host cells.^48^ Targeting RBD portions of the spike protein facilitates the detection of multiple strains of a given virus. Furthermore, the use of short peptide sequences (instead of the whole RBD or spike protein) drastically reduces reagent costs (∼1500-fold) and creates high-affinity binding sites for a specific region of the target protein, therefore enhancing the “monoclonality” of the nanoMIPs. The size and binding affinity of the nanoMIPs were initially analyzed to validate the synthesis process. Nanoparticle tracking analysis (NTA) revealed homogeneous nanoMIPs with a mean size of 68.8 ± 0.6 nm (Figure S1). Surface plasmon resonance (SPR) with the spike protein produced an equilibrium dissociation constant (*Κ*_D_) of 7 nM (Figure S2), which is comparable to SARS-CoV-2 antibodies and ACE2 receptors.^16,49−51^

**Figure 2:**
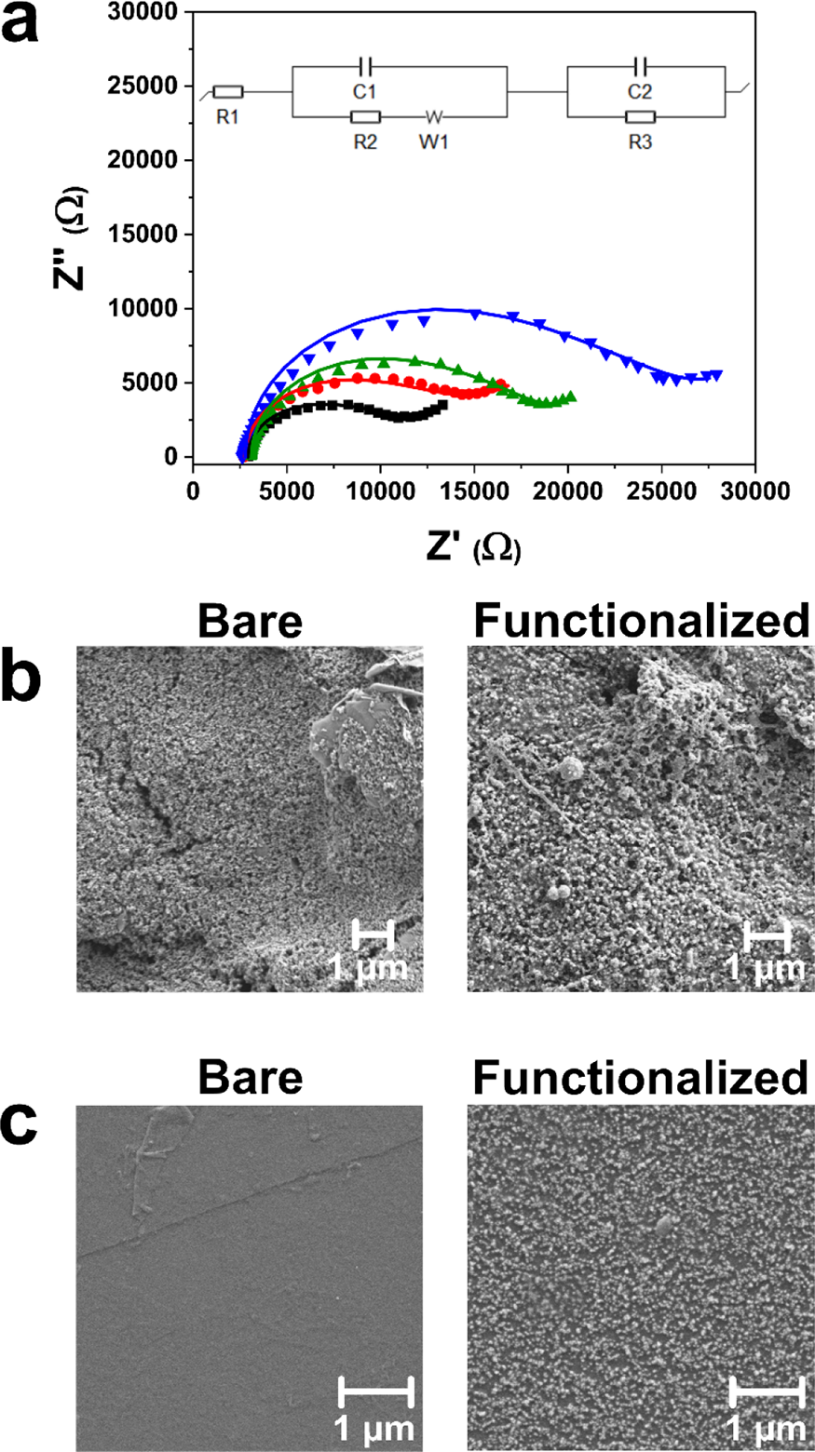
Characterization of nanoMIP-functionalized SPEs. (a) Nyquist plots for the bare SPE (black squares), 4-ABA electrografted on the SPE (red circles), activation of carboxylic groups (green triangles), and covalent coupling of nanoMIPs (blue inverted triangles). The inset shows an equivalent electrochemical circuit. Typical SEM images showing (b) bare and nanoMIP-functionalized SPEs and (c) bare and nanoMIP-functionalized HOPG surfaces.

The nanoMIPs were immobilized onto SPEs using a straightforward strategy involving electrografting of diazonium salts combined with a standard organic coupling reaction (Figure 1d). Following this procedure, electrochemical impedance spectroscopy (EIS) was utilized as a quick and straightforward technique to confirm the presence of nanoMIPs on the SPE surfaces. Nyquist plots were obtained at each stage of the covalent coupling process to monitor the change in charge-transfer resistance at the working electrode surface and thus provide an indication of nanoMIP attachment (Figure 2a). The overlaid plots clearly show an increase in charge-transfer resistance after each preparation step (total increase of 14.3 kΩ), which suggests that nanoMIPs (nonconductive) were successfully immobilized onto the SPE surfaces using the electrografting process. Scanning electron microscopy (SEM) was utilized to quantify the degree of nanoMIP immobilization on the SPEs. However, this was challenging as bare SPEs (Figure 2b) have significant surface roughness and depositions of surface binding agent, which appear similar in size to nanoMIPs. To provide further clarification, the nanoMIP immobilization procedure was repeated on freshly cleaved highly orientated pyrolytic graphite (HOPG), which acted as a model carbon-based substrate with a featureless topography. By comparing the bare and nanoMIP-functionalized HOPG (Figure 2c), it was clear that the nanoMIP surface coverage was high (mean of ∼84%) after covalent coupling. We expect this surface coverage value to be similar on the graphite SPEs, thus validating the immobilization procedure (Figure S3 presents additional images).

### Assessment of NanoMIP Robustness

**Figure 3:**
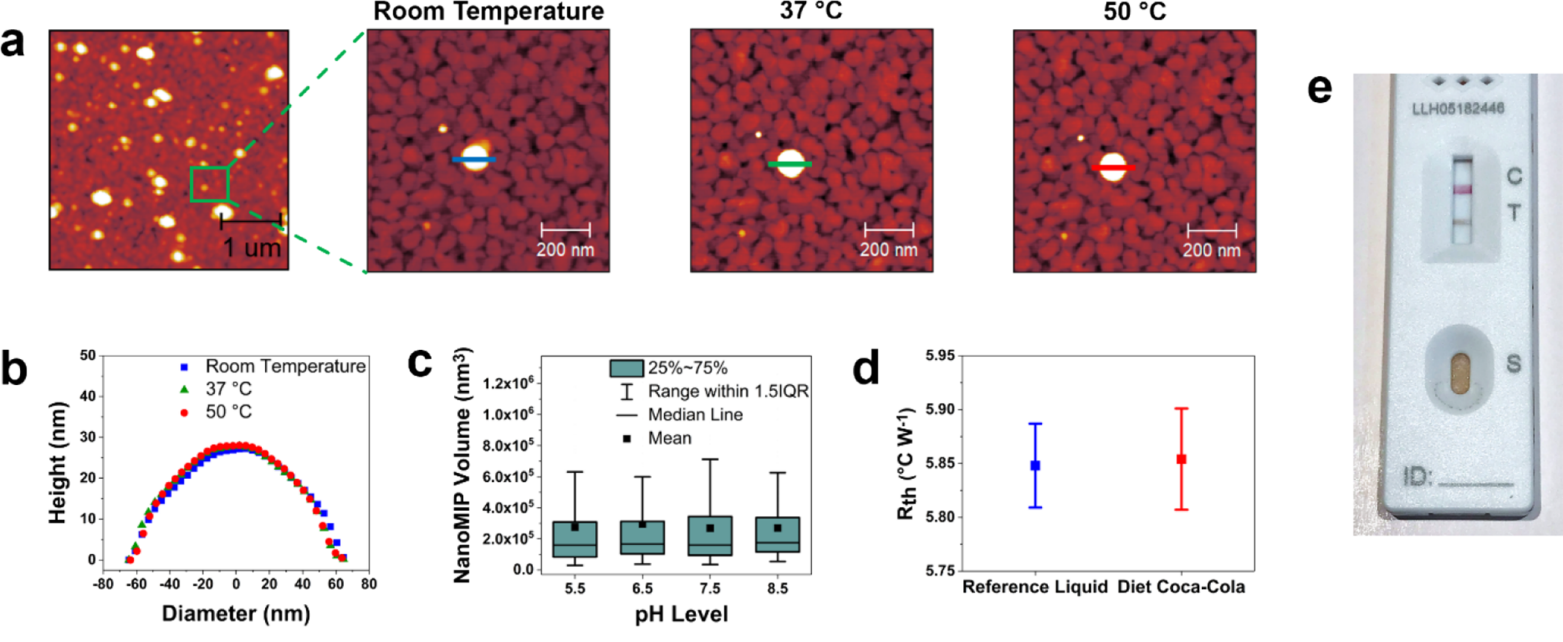
Examining the ability of nanoMIPs to withstand extremes of temperature and pH. (a) Typical AFM height images of an isolated nanoMIP on a Au surface in air (represented by the green box in the larger scale image) at room temperature, 37 °C, and 50 °C. (b) Corresponding cross-sectional profile plot of the nanoMIP at each temperature. (c) Box chart comparing nanoMIP volumes (*n* = 120) from AFM images at various pH levels. (d) Thermal response of the developed nanoMIP-based sensor to a clinical reference liquid (universal transport medium) and Diet Coca-Cola (pH = 3.5). (e) Commercial rapid antigen test giving a false positive result when Diet Coca-Cola was used as the test liquid.

The capability of nanoMIPs to withstand extremes of temperature and pH was comprehensively investigated. Atomic force microscopy (AFM) was utilized to examine how increasing temperature impacted the morphology of adsorbed nanoMIPs by imaging the same nanoMIPs at room temperature, 37 °C, and 50 °C. Typical AFM images (Figure 3a) and a corresponding cross-sectional profile plot (Figure 3b) show that the nanoMIP morphology was unaffected by increasing temperature. Moreover, tracking numerous droplets revealed minimal changes in mean nanoMIP volume (∼6% decrease) from room temperature to 50 °C. This result quantitatively confirmed that nanoMIP morphology remains consistent across relatively large temperature ranges. SPR was also performed against the spike protein after the nanoMIPs had experienced autoclaving for 85 min with a maximum temperature of 121 °C. The results revealed very similar *Κ*_D_ values for the nanoMIPs before and after autoclaving (7 and 3 nM, respectively), demonstrating that there is no impact on binding affinity after exposure to high temperatures. This is highly advantageous for the shelf-life of nanoMIPs as autoclaving (sterilization) facilitates their long-term storage in water without bacterial degradation. In contrast, antibodies experience significant deterioration in affinity at temperatures above 37 °C.^52,53^

The ability of nanoMIPs to withstand extremes of pH was also investigated using AFM by measuring the volume of adsorbed nanoMIPs (*n* = 120) in liquid at various pH levels (typical images in Figure S4). AFM image analysis revealed negligible changes to the mean nanoMIP volume from pH 5.5 to 8.5 (∼3% decrease), which highlighted that adsorbed nanoMIP morphology was consistent across a broad pH range (Figure 3c). Additionally, we compared the thermal response of the developed nanoMIP tests to a clinical reference liquid (universal transport medium, UTM) and Diet Coca-Cola (pH = 3.5). The results demonstrated that there was no statistically significant difference between the thermal responses of the two liquids (Figure 3d). In contrast, commercial antibody-based rapid antigen tests are highly impacted by changes to pH and false positive results are produced when acidic soft drinks (Diet Coca-Cola is shown in Figure 3e) are used as the test liquid (a video comparing our nanoMIP test with a commercial rapid antigen test is presented in the Supporting Information).^54^

We have demonstrated that nanoMIPs are capable of functioning in extremes of temperature and pH, which presents several critical advantages compared to biomaterial-based receptors. For example, commercial rapid antigen tests must generally be stored under strict conditions (15–30 °C) due to the temperature-sensitive nature of antibodies.^39^ Consequently, proper test storage is difficult to maintain in areas with climates that are regularly outside of these temperatures. This problem is further accentuated in low- and middle-income countries where access to temperature-controlled environments may be limited. Therefore, the temperature sensitivity of commercial antigen tests presents a serious disadvantage that may limit their use globally as well as lead to large amounts of plastic waste and/or poor diagnostic accuracy due to test spoilage. Moreover, the results highlight that nanoMIPs could potentially be used for measurements in extreme environments, such as wastewater, with limited sample pretreatment. This could be a valuable tool in monitoring and containing COVID-19 outbreaks, which are currently difficult to effectively achieve with environmentally sensitive antibody receptors due to the need for expensive and laborious pretreatment.^40^

### Thermal Detection of Antigen-Spiked Solutions

**Figure 4:**
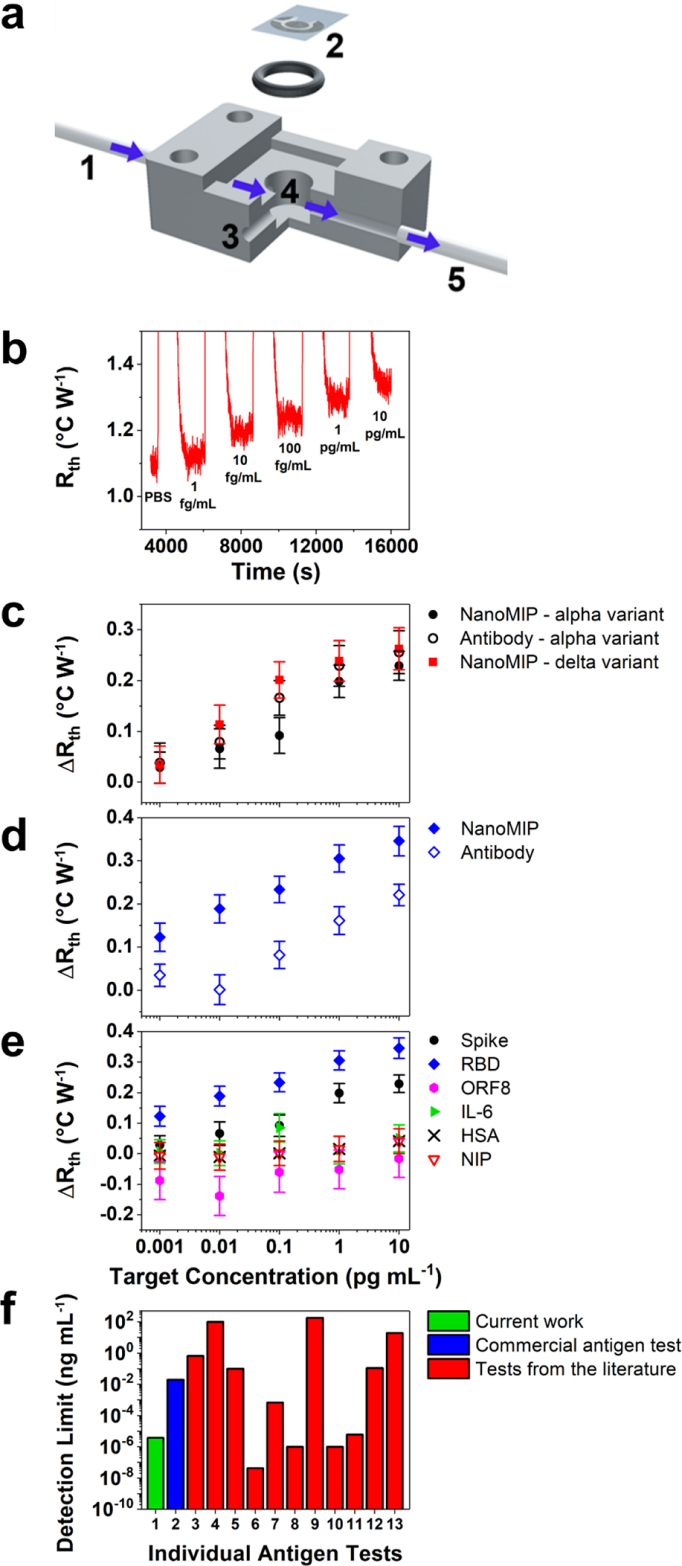
Thermal detection of antigen-spiked solutions. (a) Schematic illustration of the 3D-printed flow cell: (1) inlet tube, (2) functionalized SPE, (3) thermocouple inlet, (4) closed-bottom reservoir with an inlet/outlet to facilitate liquid flow using a syringe pump, and (5) outlet tube. (b) Typical HTM data for a nanoMIP-functionalized SPE upon exposure to PBS containing 1 fg mL^–1^ to 10 pg mL^–1^ SARS-CoV-2 RBD. Typical dose–response curves (error bars represent the SD) showing the thermal response of the (c) nanoMIP (for alpha and delta variants) and antibody sensors to the spike protein, (d) nanoMIP and antibody sensors to the RBD, and (e) nanoMIP sensors to SARS-CoV-2 antigens and the negative controls ORF8, IL-6, and HSA. The response of NIP-based sensors to the spike protein was also examined. (f) Comparing LoD values for the current work (RBD) with a commercial rapid antigen test and numerous recently developed antigen tests from the literature.

SARS-CoV-2 antigens were thermally detected by mounting nanoMIP-functionalized SPEs into 3D-printed resin flow cells (Figure 4a) to create an interface between the heat sink and the liquid reservoir. Two thermocouples measured the heat sink (*T*_1_) and liquid reservoir (*T*_2_) temperatures every second, and the thermal resistance (*R*_th_) was obtained by dividing the temperature gradient (*T*_1_–*T*_2_) over the power required to maintain the heat sink at 37.00 ± 0.02 °C. As the target attached to the nanoMIPs, heat transfer at the solid–liquid interface was reduced (larger temperature gradient), which led to a measurable increase in the *R*_th_. The injection of antigen-spiked phosphate-buffered saline (PBS) solutions was systematically performed at increasingly large concentrations (1 fg mL^–1^ to 10 pg mL^–1^) using a syringe pump. The experiments produced raw thermal data plots that displayed a stepwise increase in *R*_th_, where each stabilized plateau represents the injection of an increasingly concentrated spiked solution (Figure 4b). Dose–response curves (Figure 4c–e) were composed of the thermal plots by taking the mean and standard deviation (SD) of the stabilized plateau from each concentration injection. Limit of detection (LoD) values were calculated from the dose–response curves using the three-sigma method (3 × reference SD) in the linear range.

SARS-CoV-2 antibodies were also immobilized onto SPEs (protocol from Figure 1d) to facilitate a direct comparison between the sensing performance of nanoMIP and antibody receptors. The thermal detection results revealed that the response to the spike protein (alpha variant, Figure 4c) was very similar for the nanoMIP (LoD = 9.9 ± 2.5 fg mL^–1^) and antibody (8.9 ± 4.1 fg mL^–1^) sensors. Consequently, this highlights that the specificity of the nanoMIPs against the spike protein is comparable with antibodies used in commercial tests. The ability of nanoMIP receptors to detect virus mutations was also examined by measuring their specificity against the delta variant of the spike protein (Figure 4c). The sensor was effective in detecting the delta variant as the resulting LoD (6.1 ± 2.9 fg mL^–1^) was very similar to the value obtained for the alpha variant. This demonstrates that the nanoMIP sensor can detect equally low amounts of the alpha and delta variants of the spike protein, which is highly promising for potential commercial applications as there is ongoing concern regarding the reduction in test efficacy due to new variants.^55^ If any future variant did impact the small target epitope (∼10 amino acids of the RBD), novel nanoMIPs can be easily developed for the variant in a low-cost, safe, and rapid manner (3–4 weeks).

The versatility of nanoMIP receptors was examined by also measuring their thermal response against the RBD (Figure 4d). The LoD value for the RBD was ∼20 times smaller for the nanoMIP sensor (3.9 ± 1.0 fg mL^–1^) compared to the antibody sensor (85.5 ± 15.0 fg mL^–1^). Consequently, this demonstrates that the nanoMIPs possessed greater versatility than antibodies as they had a comparable LoD for the spike protein but a significantly lower LoD for the RBD. The selectivity of the nanoMIP sensor was also comprehensively examined using three negative controls, which are common interferents in clinical samples: open reading frame 8 (ORF8), interleukin-6 (IL-6), and human serum albumin (HSA).^16^ A high degree of binding occurred between the SARS-CoV-2 antigens and nanoMIPs, which led to large Δ*R*_th_ values at the highest concentration (10 pg mL^–1^) for the spike protein (0.23 °C W^–1^) and RBD (0.35 °C W^–1^). In contrast, minimal binding occurred with the negative controls, which led to significantly lower Δ*R*_th_ values for ORF8 (0.00 °C W^–1^), IL-6 (0.06 °C W^–1^), and HSA (0.05 °C W^–1^). The results demonstrated that the thermal response of the nanoMIP sensor was considerably greater for SARS-CoV-2 antigens compared to the negative controls, which highlighted the excellent selectivity of the nanoMIP sensor. An additional control experiment was performed using non-imprinted polymers (NIPs) immobilized to SPEs (protocol from Figure 1d). NIPs were prepared using the same synthesis protocol as nanoMIPs, except that they were not exposed to a target epitope during polymerization and therefore had no specific cavities to facilitate binding with SARS-CoV-2. The thermal response of a nanoMIP-functionalized SPE to the spike protein was ∼6 times larger compared to the NIP-functionalized SPE (0.04 °C W^–1^). Subsequently, this shows that specific binding occurred between the spike protein and nanoMIP cavities.

The LoD values of our nanoMIP sensor (for the RBD), a commercial rapid antigen test, and numerous recently developed antigen tests from the literature are presented in Figure 4f.^41−47^ Additional information on each test can be found in Table S1 (corresponds to *x*-axis labels in Figure 4f). Commercial rapid antigen tests often possess sensitivities that fail to meet the required standards of the WHO, which present a significant obstacle for effective screening of the general population.^13,14^ The LoD value for our nanoMIP sensor is ∼6000 times lower than the commercial rapid antigen test (20 pg mL^–1^). This considerably lower LoD highlights that nanoMIP receptors could be a valuable tool in producing rapid antigen tests with adequate sensitivities for effective population screening. Additionally, the LoD of the nanoMIP sensor is among the lowest values from recently developed antigen tests in the literature. This demonstrates that thermal detection using nanoMIPs can compete with the best performing antigen tests from the recent literature while possessing the added benefit of being able to withstand extremes of temperature and pH. An interesting avenue for future research would be to consider incorporating a redox probe into the nanoMIP structure, which would enable simultaneous electrochemical and thermal detection.

### Thermal Detection of Clinical Samples

**Figure 5:**
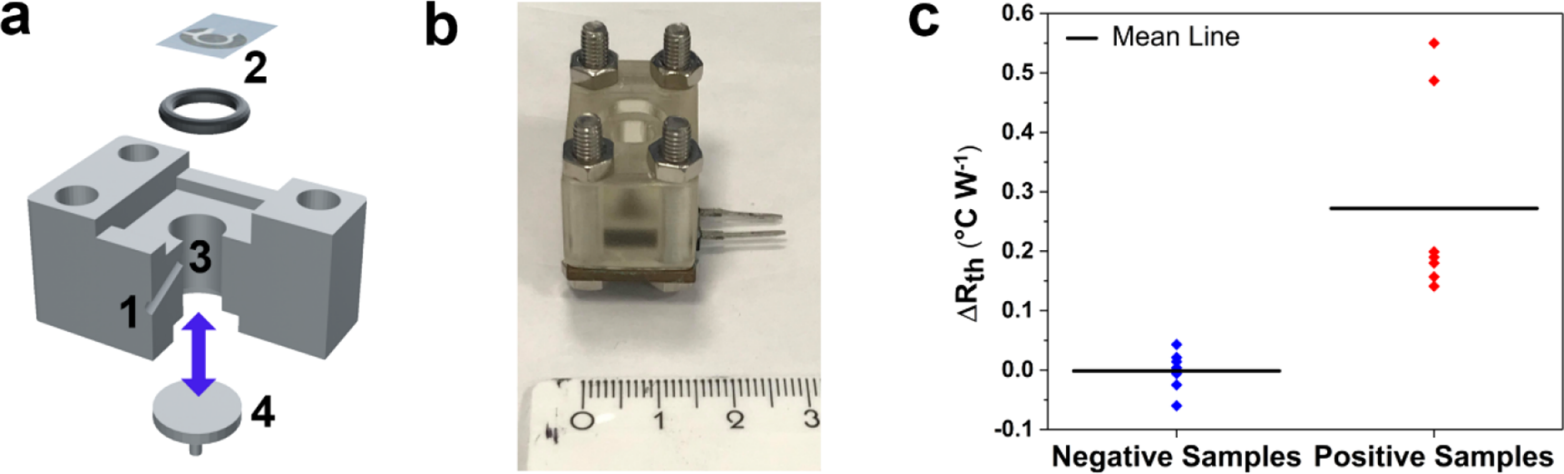
Thermal detection of clinical samples. (a) Schematic illustration of the 3D-printed prototype addition cell: (1) thermocouple inlet, (2) functionalized SPE, (3) open-bottom reservoir to facilitate the manual addition of liquid, and (4) removable lid to reduce experimental noise. (b) Photograph of the addition cell. (c) Thermal response of the nanoMIP sensor to clinical samples from COVID-positive and COVID-negative patients (*n* = 7).

A prototype 3D-printed resin cell (addition cell, Figure 5a,b) was developed for clinical analysis with disposable components. In this prototype, the test liquid was simply added with a pipette, which resulted in less disturbance to the system (*e.g.*, flow and addition of air bubbles) compared to using a syringe pump. This is highly advantageous for clinical analysis since the sample volume was similar to that collected by a throat and nasal swab (100 μL), the measurement time was reduced to ∼15 min, and device operation was straightforward. To validate the addition cell design, thermal detection experiments were performed using the spike protein in PBS. The addition cell sensor showed a good thermal response to the spike protein (Figure S5) as the LoD value (7.0 ± 4.0 fg mL^–1^) was very similar to the results obtained when using the flow cell design (9.9 ± 2.5 fg mL^–1^).

After initial validation, clinical measurements were performed using COVID-positive (cycle threshold, <20 cycles) and COVID-negative patient samples (*n* = 7). The thermal detection results (Figure 5c) show that specific binding to the nanoMIP cavities occurred during measurements of the positive samples, which led to a large mean Δ*R*_th_ value (0.27 °C W^–1^). In contrast, only nonspecific binding occurred to the nanoMIPs when measuring the negative samples, which resulted in a mean Δ*R*_th_ of 0.00 °C W^–1^. The overall range of Δ*R*_th_ values was much larger for the positive samples (0.41 °C W^–1^) compared to the negative samples (0.10 °C W^–1^), which is due to variations in the viral loads of different COVID-positive patients. Importantly, there was no overlap in any positive and negative measurements as the largest Δ*R*_th_ for a negative sample was ∼4 times lower than the smallest Δ*R*_th_ for a positive sample. This highlights that the nanoMIP sensor possesses excellent sensitivity and specificity for the detection of SARS-CoV-2 in clinical samples. Furthermore, the measurement time of the nanoMIP sensor (∼15 min) is comparable to commercial rapid antigen tests, which is crucially important for potential population screening applications.

## Conclusions

We developed a novel sensor for the thermal detection of SARS-CoV-2 using nanoMIPs electrografted onto low-cost SPEs fitted inside 3D-printed measurement cells. We demonstrate that, unlike antibodies and other biomaterial-based receptors, nanoMIPs can withstand extremes of temperature and pH without experiencing deterioration in sensing performance. The nanoMIP sensor exhibited a similar LoD for the spike protein (∼9.9 fg mL^–1^) and a significantly lower LoD for the RBD (∼3.9 fg mL^–1^) compared to an antibody sensor. Furthermore, the nanoMIP sensor displayed excellent selectivity against three clinically relevant negative controls, as well as the ability to detect equally low amounts of the alpha and delta variants of the spike protein. The obtained LoD values are ∼6000 times lower than a commercial rapid antigen test and among the lowest from antigen tests in recent literature reports. Clinical measurements using a prototype 3D-printed addition cell rapidly detected (∼15 min) SARS-CoV-2 in patient samples with excellent sensitivity and specificity. The developed nanoMIP sensor possesses the unique combination of exceptional sensing performance and the ability to withstand extreme environmental conditions. This creates significant commercial potential for nanoMIP tests that can accurately detect SARS-CoV-2 while offering an extended shelf-life and less stringent storage conditions compared to antibody-based tests. Furthermore, there is potential for nanoMIPs to be utilized for measurements in wastewater and other diagnostically challenging environments without the need for laborious and expensive sample pretreatment, which is currently required for tests with environmentally sensitive antibody receptors.

## Experimental Section

### Reagents

Peptide sequences employed in nanoMIP development (Ontores Biotechnology, Zhejiang, China), monomers for nanoMIP synthesis (Sigma, Gillingham, UK), sodium hydroxide (Sigma), ammonium persulfate (Sigma), mercaptoundecanoic acid (Sigma), ethanolamine hydrochloride (Sigma), bovine serum albumin (BSA, Sigma), Pluronic solution (Sigma), 4-ABA (Fisher Scientific, Loughborough, UK), EDC (Fisher Scientific), NHS (Fisher Scientific), ferricyanide (Sigma), ferrocyanide (Sigma), potassium chloride (KCl, Sigma), hydrogen peroxide (30%, Sigma), ammonia solution (25%, VWR International, Leicestershire, UK), PBS tablets (Sigma), the alpha variant of the SARS-CoV-2 spike protein (The Native Antigen Company, Kidlington, UK), the delta variant of the spike protein (Abbexa, Cambridge, UK), HSA (Sigma), and IL-6 (Bio-Rad, Watford, UK) were used as received. The SARS-CoV-2 RBD and ORF8 were provided by the Medical Research Council Protein Phosphorylation and Ubiquitylation Unit (Dundee, UK). PBS solutions were prepared with deionized (DI) water, and AFM experiments were performed using Milli-Q water (both with a resistivity of ≥18.2 MΩ cm).

### Clinical Sample Preparation

The clinical samples were diagnostic remnants collected from patients symptomatic for COVID-19 infection as part of registered protocols approved by the Research Ethics Committee of North East–Newcastle and Tyneside 1 (REC reference 17/NE/0070). SARS-CoV-2 was subsequently detected from combined nose and throat swabs stored in IMPROVIRAL viral preservation medium (VPM, Scientific Laboratory Supplies, Nottingham, UK) at the Integrated Covid Hub North East (ICHNE) Lighthouse Laboratory (Gateshead, UK). Viral gene expression was determined using the Thermo Fisher Scientific Amplitude Solution automated real-time PCR system and TaqPath COVID-19 high-throughput detection assay (Thermo Fisher Scientific, Paisley, UK), followed by heat deactivation at 65 °C for 30 min. Residual samples were then stored at −80 °C within the ICHNE Innovation Laboratory Biobank for future use. Negative samples were obtained from healthy volunteers with confirmed negative rapid antigen tests. Thermal measurements were performed immediately after collection where the negative samples were processed with UTM from rapid antigen test kits (Xiamen Biotime Biotechnology Co. Ltd., Fujian, China).

### NanoMIP Synthesis and Characterization

The synthesis method was adapted from our previous work in which glass beads (70–100 μm in diameter) were used as a solid support for the immobilization of the target molecule.^22^ The beads were first activated with sodium hydroxide (4 M) and then functionalized with an amino-silane to obtain free amine groups on their surface. Epitopes of the RBD region were identified by *in silico* analysis. These epitopes were then produced and immobilized onto the amine-derivatized glass beads via succinimidyl-iodoacetate coupling. Immobilization of the peptide was confirmed by monitoring color changes with a bicinchoninic acid assay. Polymerization was then initiated by ammonium persulfate (Figure 1a). After polymerization (1 h), the solid support was used to isolate high-affinity nanoMIPs from the remaining monomers, oligomers, and low-affinity polymers. This was achieved via a low-temperature elution (20 °C, Figure 1b), followed by an elevated temperature elution (60 °C, Figure 1c). NIPs were synthesized using the same protocol, but they were not exposed to the target epitope during polymerization. Screening experiments were performed using three types of nanoMIPs with different monomer compositions and target peptides. The nanoMIP that exhibited the lowest initial LoD values for the SARS-CoV-2 antigens was selected for all experiments within the paper. Typical dose–response curves for the other two nanoMIP types are presented in Figure S6.

NTA was performed using a NanoSight NS300 (NanoSight Ltd., Malvern, UK) equipped with NanoSight NTA 3.4 software. Five independent analyses were performed at room temperature (22.8 °C). Prior to analysis, the samples were dialyzed using a SnakeSkin (Fisher Scientific) dialysis membrane (molecular weight cut-off, 10 kDa), diluted in DI water to a concentration of ∼10^8^ particles mL^–1^, and sonicated for 2 min. SPR analysis was performed on a Biacore 3000 (Cytiva, Sheffield, UK). SIA Au chips (Cytiva) were modified with mercaptoundecanoic acid before the spike protein (300 nM) was coupled to them via EDC/NHS chemistry. Excess NHS esters were deactivated by injecting 100 μL of ethanolamine hydrochloride (0.1 M) at 10 μL min^–1^, followed by a 1% BSA/Pluronic solution. Flow conditions were set at 30 μL min^–1^, and a control channel functionalized with BSA was used as a negative control. Five different concentrations of the nanoMIPs (1.25–20 nM) were injected in PBS. The dissociation time was set at 5 min, and *Κ*_D_ values were obtained using BiaEvaluation software (v 4.1).

### Electrode Functionalization and Characterization

The graphite SPEs (3.1 mm in diameter) were produced by screen-printing a graphite ink formulation (Gwent Electronic Materials Ltd., Monmouthshire, UK) onto a standard polyester substrate.^56^ This was followed by curing at 60 °C for 30 min with a dielectric material (Gwent Electronic Materials Ltd.), which was used to define the rectangular shape of the SPE for easy handling.^57^ The experimental protocol for the electrografting procedure (Figure 1d) is comprehensively outlined in our previous work.^34^ SARS-CoV-2 antibody-functionalized SPEs and NIP-functionalized SPEs were prepared according to the same electrografting protocol.

EIS measurements were performed on a PalmSens4 potentiostat (PalmSens, Houten, The Netherlands) in PBS with ferricyanide (1 mM), ferrocyanide (1 mM), and KCl (0.1 M) with a fixed frequency range of 0.1 Hz to 100 kHz. An Ag/AgCl reference electrode and Pt counter electrode (Alvatek Ltd., Romsey, UK) were used for the measurements. SEM images were obtained using a Supra 40VP field emission scanning electron microscope (Carl Zeiss Ltd., Cambridge, UK). Before imaging, SPEs or HOPG substrates (NT-MDT SI, Moscow, Russia) were coated with a thin layer of Au/Pd (8 V, 30 s) using a SCP7640 coater (Polaron, Hertfordshire, UK). NanoMIP surface coverage on the HOPG was obtained from SEM images using the freeware Gwyddion (v 2.59).

AFM measurements were performed on a JPK Nanowizard 4 XP Bioscience (Bruker, Nano GmbH, Berlin, Germany). Measurements in air were carried out in tapping mode using PPP-NCL-W probes (Nanosensors, Neuchatel, Switzerland) with a cantilever length of ∼225 μm and a spring constant of ∼48 N m^–1^. Measurements in liquid were performed in quantitative imaging (QI) mode using MLCT-E probes (Bruker, Ca, USA) with a cantilever length of ∼140 μm and a spring constant of ∼0.1 N m^–1^. Au-coated Si chips were used as substrates (Si-Mat, Kaufering, Germany). Prior to drop-casting, the chips were cleaned by immersion for 5 min in a 5:1:1 mixture of Milli-Q water, ammonia, and hydrogen peroxide heated at 75 °C. The chips were then rinsed in Milli-Q water and dried with nitrogen. NanoMIP solutions were diluted in Milli-Q water to ∼2.54 μg mL^–1^, drop-cast (20 μL) onto the Au-coated surfaces, and allowed to dry in ambient conditions for a minimum of 4 h in a Petri dish. A high-temperature heating stage (HTHS, JPK BioAFM; resolution of 0.1 °C) was used as a temperature controller to facilitate imaging at 30 and 50 °C. QI measurements were performed at room temperature (23 ± 1 °C) in liquid at different pH levels (5.5–8.5) within the operating conditions of AFM. Initial measurements were carried out in pure Milli-Q water (pH 5.5), and the pH was subsequently increased with the addition of ammonia. The nanoMIP volume was calculated using Gwyddion with previously established methods (eq S1).^58,59^

### Thermal Measurements

Flow and addition cells (Figures 4a and 5a) were 3D-printed using an Anycubic Photon printer (Shenzhen, China). For all experiments, the thermal measurement device was controlled using LabView software and a proportional-integral-derivative (PID) controller attached to a power resistor (22 Ω) regulated the feedback on the signal.^60^ The PID parameters were optimized to reduce noise and were set at *P* = 1, *I* = 13, and *D* = 0.2 for the flow cell experiments and *P* = 1, *I* = 14, and *D* = 0 for the addition cell experiments.

For the flow cell experiments (Figure 4), the liquid reservoir was filled with PBS and left for 30 min to ensure stabilization of the baseline *R*_th_ signal. Subsequently, five spiked PBS solutions (3 mL) with increasing concentrations of the target (1 fg mL^–1^ to 10 pg mL^–1^) were injected into the flow cell at a rate of 250 μL min^–1^ for 12 min using an automated syringe pump (LSP02-1B, Longer Precision Pump Co., Hebei, China). The system was allowed to stabilize for 30 min prior to each subsequent injection. All measurements were performed in triplicate. For the addition cell experiments (Figure 5), UTM and VPM were used as reference liquids for the negative and positive samples, respectively. During the measurements, 100 μL of the reference liquid (UTM/VPM) was pipetted into the reservoir and the *R*_th_ signal was allowed to stabilize for 10 min. Following this, the reference liquid was pipetted out and 100 μL of the sample was added.

## Supporting Information Available

NTA spectrum; SPR spectrum; additional SEM images; typical AFM images of nanoMIPs at various pH levels; comparison of different SARS-CoV-2 antigen tests; typical dose–response curve for addition cell experiments with antigen-spiked solutions; typical dose–response curves for additional nanoMIP types; nanoMIP volume equation (PDF)
Video comparing the responses of our nanoMIP test and a commercial rapid antigen test to Diet Coca-Cola (MOV)

## Supplementary Material

se2c00100_si_001.pdf

se2c00100_si_002.mov
