# Supplementary material for: Molecularly Imprinted Polymer Nanoparticles Enable Rapid, Reliable, and Robust Point-of-Care Thermal Detection of SARS-CoV-2
Source: ACS Sens. 2022 Apr 13;7(4):1122–31. doi: 10.1021/acssensors.2c00100 (PMC9016778; doi:10.1021/acssensors.2c00100)
Supplement: Supplementary file 1 — se2c00100_si_001.pdf [file se2c00100_si_001.pdf]

Supporting Information

Molecularly Imprinted Polymer Nanoparticles Enable Rapid, Reliable, and Robust Point-of-care Thermal Detection of SARS-CoV-2

Jake McClements[1], Laure Bar[2], Pankaj Singla[1], Francesco Canfarotta[3,*], Alan Thomson[3], Joanna Czulak[3], Rhiannon E. Johnson[3], Robert D. Crapnell[4], Craig E. Banks[4], Brendan Payne[5,6], Shayan Seyedin[1], Patricia Losada-Pérez[2], and Marloes Peeters[1,*]

1) Newcastle University, School of Engineering, Merz Court, Claremont Road, NE1 7RU, Newcastle Upon Tyne, UK.

2) Experimental Soft Matter and Thermal Physics (EST) group, Department of Physics, Université Libré de Bruxelles, Boulevard du Triomphe CP223, 1050 Brussels, Belgium.

3) MIP Diagnostics Ltd., The Exchange Building, Colworth Park, Sharnbrook, MK44 1LQ, Bedford, UK.

4) Manchester Metropolitan University, Faculty of Science and Engineering, John Dalton Building, Chester Street, M1 5GD, Manchester, UK.

5) Newcastle-upon-Tyne Hospitals NHS Foundation Trust, Department of Infection and Tropical Medicine, Royal Victoria Infirmary, Newcastle-upon-Tyne, NE1 4LP, UK.

6) Newcastle University, Translational and Clinical Research Institute, Medical School, Framlington Place, Newcastle-upon-Tyne, NE1 7RU, UK.

Corresponding authors*: Marloes Peeters for thermal detection/Francesco Canfarotta for nanoMIP synthesis.

1. NanoMIP size analysis

The size of the molecularly imprinted polymer nanoparticles (nanoMIPs) was obtained using nanoparticle tracking analysis (NTA) at room temperature (Figure S1). The average particle size was 68.8 ± 0.6 nm (mode peak at 54.9 ± 2.5 nm).

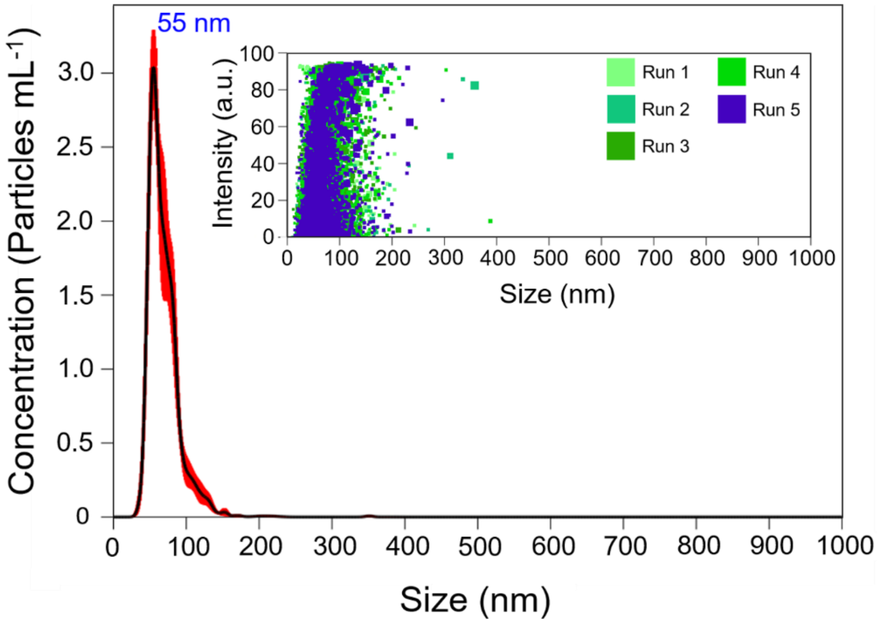

Figure S1. NTA spectrum for the nanoMIPs in deionized water. The insert presents the intensity vs size graph, which highlights the individual events at larger sizes for the five replicates.

2. Surface plasmon resonance analysis

Surface plasmon resonance (SPR) was performed to assess nanoMIP binding affinity to the SARS-CoV-2 spike protein (Figure S2). The SPR analysis produced an equilibrium dissociation constant (K_D) of 7 nM.

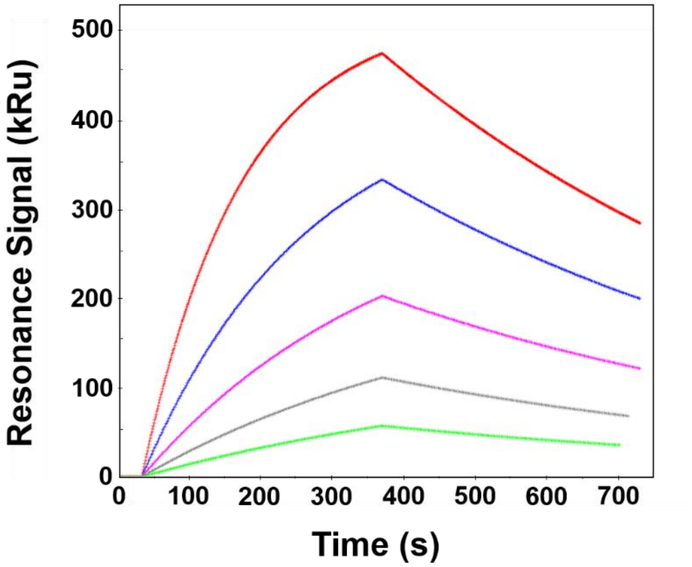

Figure S2. SPR spectrum for the nanoMIPs with the SARS-CoV-2 spike protein.

3. Scanning electron microscopy characterization

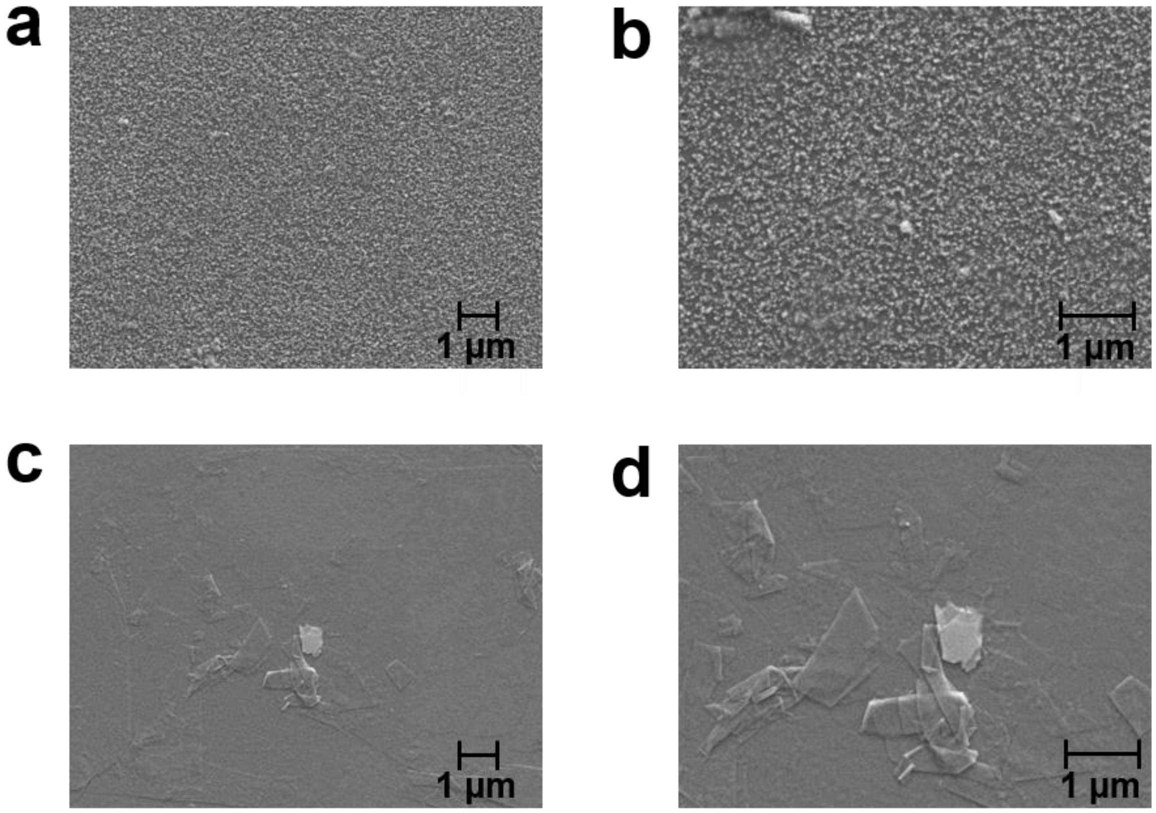

Figure S3. Scanning electron microscopy (SEM) images of nanoMIP-functionalized highly orientated pyrolytic graphite (HOPG) substrates at **a)** 20 kX and **b)** 40 kX magnification, in addition to bare HOPG substrates (reference) at **c)** 20 kX and **d)** 40 kX magnification.

4. Atomic force microscopy characterization

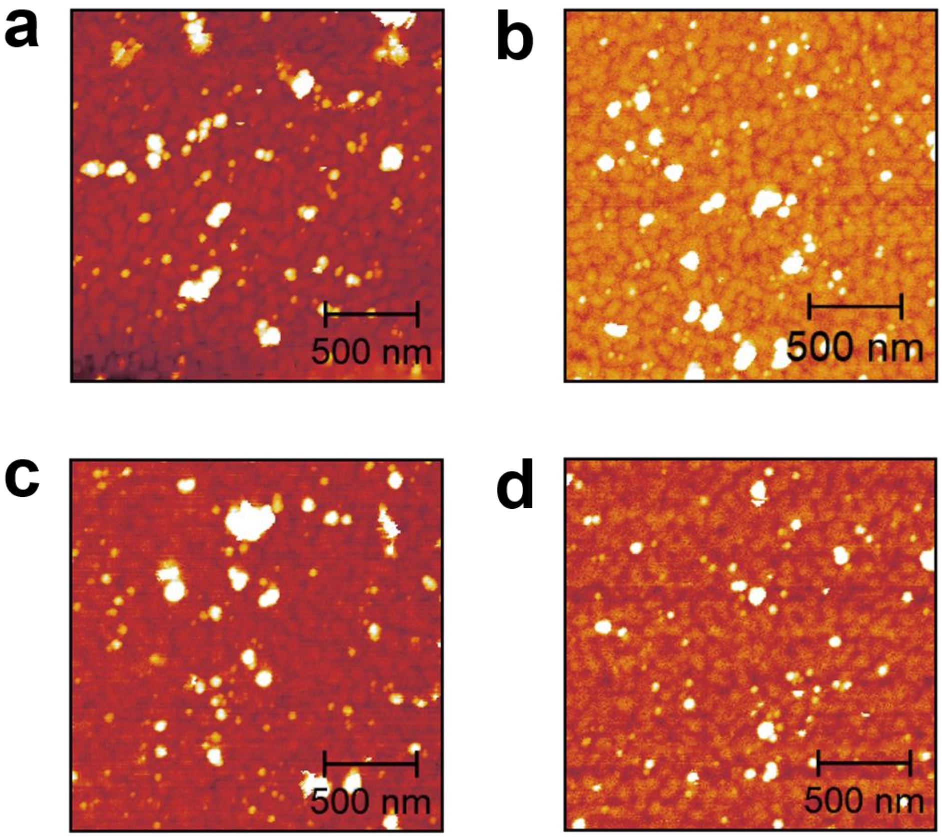

Figure S4. Typical atomic force microscopy (AFM) images of nanoMIPs on Au surfaces in liquid with pH levels of **a)** 5.5, **b)** 6.5, **c)** 7.5, and **d)** 8.5.

5. Comparison with other antigens tests

Table S1. Comparison of different SARS-CoV-2 antigen tests.

Biosensor[a]	Method[b]	Analyte[c]	Limit of detection	Label in Figure 4f	Ref
NanoMIP-based thermal sensor	NanoMIPs immobilized onto SPEs. HTM used for detection.	RBD and spike protein	RBD: 3.9 fg mL^{-1} S protein: 9.9 fg mL^{-1}	1	Current work
WANTAI SARS-CoV-2 Ag Rapid Test LFA	Lateral flow immunochromatography combined with double antibody sandwich method.	Nucleocapsid protein	20 pg mL^{-1}	2	1
Half-strip LFA	Test strip with immobilized antibodies and optical read out.	Nucleocapsid protein	0.65 ng mL^{-1}	3	2
Fluorescent microsphere LFA biosensor	Test strip combining fluorescent microsphere labelling and immunochromatography technology.	Nucleocapsid protein	100 ng mL^{-1}	4	3
Nanozyme CL paper test	Nanozyme and enzymatic CL immunoassay combined with a lateral flow strip.	Spike protein	100 pg mL^{-1}	5	4
Electrochemical immunosensor with Cu_2O nanocube coating	Spike antibodies immobilized onto SPEs modified with Cu_2O nanocubes. CV and EIS used for detection.	Spike protein	0.04 fg mL^{-1}	6	5
MIP-based electrochemical sensor	Disposable Au-TFE chip modified with ncovNP-MIPs. DPV used for detection.	Nucleocapsid protein	0.7 pg mL^{-1}	7	6
FET-based biosensor	Electrical measurements using graphene-based FET functionalized with spike antibodies.	Spike protein	1 fg mL^{-1}	8	7
Colorimetric assay using AuNPs	AuNPs capped with thiol-modified ASOs specific for nucleocapsid protein. SPR used for detection. Addition of RNase H produces a precipitate for visual detection.	Nucleocapsid protein	180 ng mL^{-1}	9	8
Cell-based biosensor	Mammalian Vero cells engineered by electroinsertion of spike antibodies. Change in bioelectric properties measured	Spike protein	1 fg mL^{-1}	10	9

	with a cell-biosensor set up using the principles of BERA.				
SERS-based biosensor	AuNPs immobilized onto Si wafer to form SERS-active substrate with spike antibodies attached. Raman reporter-labelled immuno-AgNPs used as SERS nanotags for detection.	Spike protein	6 fg mL^{-1}	11	10
ePAD	Spike antibody immobilized onto ePAD surface. SWV used for detection.	Spike protein	110 pg mL^{-1}	12	11
Magnetic bead-based electrochemical immunosensor	Magnetic beads used as support for immunological chain and combined with a carbon black-based SPE. DPV used for detection.	Spike and nucleocapsid proteins	S protein: 19 ng mL^{-1}<br>N protein: 8 ng mL^{-1}	13	12

[a] Lateral flow assay (LFA); Chemiluminescence (CL); Molecularly imprinted polymer (MIP); Field-effect transistor (FET); Gold nanoparticles (AuNPs); Surface enhanced Raman scattering (SERS); Electrochemical paper-based analytical device (ePAD).

[b] Screen-printed electrode (SPE); Heat transfer method (HTM); Cyclic voltammetry (CV); Electrochemical impedance spectroscopy (EIS); Gold-thin film electrode (Au-TFE); Molecularly imprinted polymer with selectivity for the SARS-CoV-2 nucleocapsid protein (ncovNP-MIP); Differential pulse voltammetry (DPV); Antisense oligonucleotide (ASO); Surface plasmon resonance (SPR); Ribonuclease H (RNase H); Bioelectric recognition assay (BERA); Silver nanoparticles (AgNPs); Square wave voltammetry (SWV).

[c] Receptor binding domain (RBD).

6. Thermal data from antigen experiments with addition cell set up

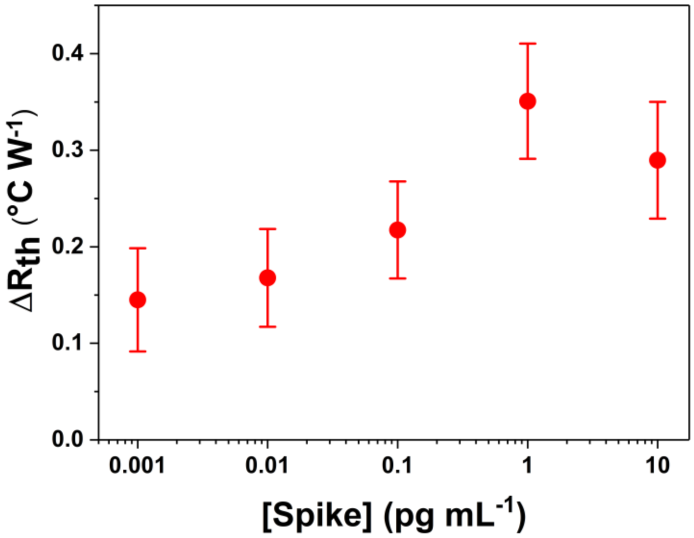

Figure S5. Typical dose-response curve when using the prototype addition cell for the thermal detection of the spike protein (1 fg mL^{-1} – 10 pg mL^{-1}) in phosphate-buffered saline (PBS).

7. Thermal data from additional nanoMIP types

Screening experiments were initially performed using three different SARS-CoV-2 nanoMIP types, named 'nanoMIP types 1-3'. The nanoMIPs differed from one another in terms of their monomer compositions and target peptides. The nanoMIP with optimal performance (nanoMIP type 1) was selected for all the main experiments. Figure S6 presents typical dose-response curves for the other two nanoMIP types (nanoMIP type 2 and nanoMIP type 3) against the spike protein and RBD.

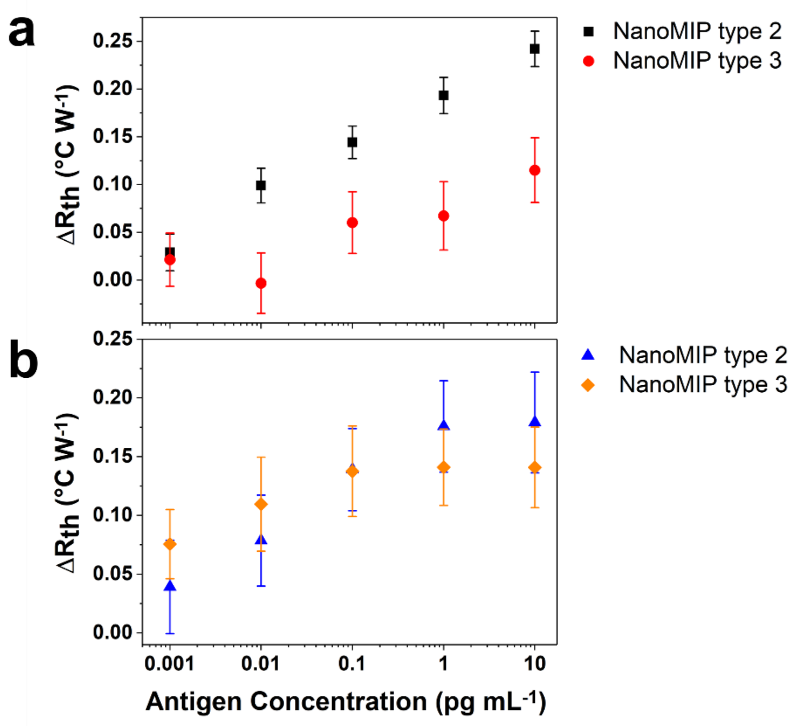

Figure S6. Typical dose-response curves against **a)** spike protein and **b)** RBD spiked PBS solutions (1 fg mL^{-1} – 10 pg mL^{-1}) for the two nanoMIP types which exhibited inferior performance compared to those used in the manuscript.

8. NanoMIP volume equation

The volume (V_{cap}) of the spherical cap-shaped adsorbed nanoMIPs was calculated using the following equation where h is nanoMIP height and r is nanoMIP radius:

$$V_{cap} = \frac{1}{6}\pi h(3r^2 + h^2) \qquad \text{(S1)}$$

References

(1) Diagnostics Wantai SARS-CoV-2. *WANTAI SARS-CoV-2 Ag Rapid Test (Colloidal Gold)*; 2020.

(2) Grant, B. D.; Anderson, C. E.; Williford, J. R.; Alonzo, L. F.; Glukhova, V. A.; Boyle, D. S.; Weigl, B. H.; Nichols, K. P. SARS-CoV-2 Coronavirus Nucleocapsid Antigen-Detecting Half-Strip Lateral Flow Assay toward the Development of Point of Care Tests Using Commercially Available Reagents. *Anal. Chem.* **2020**, *92*, 11305–11309.

(3) Zhang, C.; Zhou, L.; Du, K.; Zhang, Y.; Wang, J.; Chen, L.; Lyu, Y.; Li, J.; Liu, H.; Huo, J.; Li, F.; Wang, J.; Sang, P.; Lin, S.; Xiao, Y.; Zhang, K.; He, K. Foundation and Clinical Evaluation of a New Method for Detecting SARS-CoV-2 Antigen by Fluorescent Microsphere Immunochromatography. *Front. Cell. Infect. Microbiol.* **2020**, *10*, 10.3389/fcimb.2020.553837.

(4) Liu, D.; Ju, C.; Han, C.; Shi, R.; Chen, X.; Duan, D.; Yan, J.; Yan, X. Nanozyme Chemiluminescence Paper Test for Rapid and Sensitive Detection of SARS-CoV-2 Antigen. *Biosens. Bioelectron.* **2021**, *173*, 10.1016/j.bios.2020.112817.

(5) Rahmati, Z.; Roushani, M.; Hosseini, H.; Choobin, H. Electrochemical Immunosensor with Cu2O Nanocube Coating for Detection of SARS-CoV-2 Spike Protein. *Microchim. Acta* **2021**, *188*, 10.1007/s00604-021-04762–04769.

(6) Raziq, A.; Kidakova, A.; Boroznjak, R.; Reut, J.; Öpik, A.; Syritski, V. Development of a Portable MIP-Based Electrochemical Sensor for Detection of SARS-CoV-2 Antigen. *Biosens. Bioelectron.* **2021**, *178*, 10.1016/j.bios.2021.113029.

(7) Seo, G.; Lee, G.; Kim, M. J.; Baek, S. H.; Choi, M.; Ku, K. B.; Lee, C. S.; Jun, S.; Park, D.; Kim, H. G.; Kim, S. J.; Lee, J. O.; Kim, B. T.; Park, E. C.; Kim, S. Il. Rapid Detection of COVID-19 Causative Virus (SARS-CoV-2) in Human Nasopharyngeal Swab Specimens Using Field-Effect Transistor-Based Biosensor. *ACS Nano* **2020**, *14*, 5135–5142.

(8) Moitra, P.; Alafeef, M.; Alafeef, M.; Alafeef, M.; Dighe, K.; Frieman, M. B.; Pan, D.; Pan, D.; Pan, D. Selective Naked-Eye Detection of SARS-CoV-2 Mediated by N Gene

Targeted Antisense Oligonucleotide Capped Plasmonic Nanoparticles. *ACS Nano* **2020**, *14*, 7617–7627.

(9) Mavrikou, S.; Moschopoulou, G.; Tsekouras, V.; Kintzios, S. Development of a Portable, Ultra-Rapid and Ultra-Sensitive Cell-Based Biosensor for the Direct Detection of the SARS-CoV-2 S1 Spike Protein Antigen. *Sensors* **2020**, *20*, 10.3390/s20113121.

(10) Zhang, M.; Li, X.; Pan, J.; Zhang, Y.; Zhang, L.; Wang, C. Ultrasensitive Detection of SARS-CoV-2 Spike Protein in Untreated Saliva Using SERS-Based Biosensor. *Biosens. Bioelectron.* **2021**, *190*, 10.1016/j.bios.2021.113421.

(11) Yakoh, A.; Pimpitak, U.; Rengpipat, S.; Hirankarn, N.; Chailapakul, O.; Chaiyo, S. Paper-Based Electrochemical Biosensor for Diagnosing COVID-19: Detection of SARS-CoV-2 Antibodies and Antigen. *Biosens. Bioelectron.* **2021**, *176*, 10.1016/j.bios.2020.112912.

(12) Fabiani, L.; Saroglia, M.; Galatà, G.; De Santis, R.; Fillo, S.; Luca, V.; Faggioni, G.; D’Amore, N.; Regalbuto, E.; Salvatori, P.; Terova, G.; Moscone, D.; Lista, F.; Arduini, F. Magnetic Beads Combined with Carbon Black-Based Screen-Printed Electrodes for COVID-19: A Reliable and Miniaturized Electrochemical Immunosensor for SARS-CoV-2 Detection in Saliva. *Biosens. Bioelectron.* **2021**, *171*, 10.1016/j.bios.2020.112686.
